# Erythropoietin promotes the differentiation of fetal neural stem cells into glial cells via the erythropoietin receptor‐β common receptor/Syne‐1/H3K9me3 pathway

**DOI:** 10.1111/cns.13876

**Published:** 2022-06-17

**Authors:** Zhen‐Hong Yang, Si‐Jia Zhang, Hai‐Ping Zhao, Fang‐Fang Li, Zhen Tao, Yu‐Min Luo, Rong‐Liang Wang

**Affiliations:** ^1^ Institute of Cerebrovascular Disease Research Xuanwu Hospital of Capital Medical University Beijing China; ^2^ Beijing Geriatric Medical Research Center and National Clinical Research Center for Geriatric Disorders Beijing China; ^3^ Beijing Institute for Brain Disorders Capital Medical University Beijing China

**Keywords:** erythropoietin, hypoxia, neural stem and progenitor cells, β common receptor

## Abstract

**Aims:**

To investigate the effect of erythropoietin (EPO) on the differentiation of neural stem cells (NSCs)/neural progenitors (NPs) in the treatment of hypoxic–ischemic injury and its potential mechanisms.

**Methods:**

Fetal NSCs/NPs were treated with EPO after oxygen and glucose deprivation/reoxygenation (OGD/R). Cell viability, proliferation, and differentiation of NSCs/NPs were detected by CellTiter‐Glo, Edu assay, flow cytometry, and quantitative real‐time PCR (qPCR). Immunofluorescence staining, co‐immunoprecipitation (Co‐IP), and western blotting were used to test the existence of EPO receptor/β common receptor (EPOR/βCR) heterodimer on NSCs/NPs and the possible pathway.

**Results:**

EPO treatment at different time points increased cell viability without affecting proliferation. EPO treatment immediately after OGD/R promoted oligodendrocyte and astrocyte differentiation, while decreasing neuronal differentiation of NSCs/NPs. EPOR/βCR heterodimer existed on the cell surface of the fetal cortical NSCs/NPs, EPO treatment significantly increased the mRNA expression of βCR and elevated the correlation between EPOR and βCR levels. In addition, mass spectrometry analysis identified Syne‐1 as a downstream signaling molecule of the EPOR/βCR heterodimer. Immunofluorescence staining and western blotting indicated that the βCR/Syne‐1/H3K9me3 pathway was possibly involved in the differentiation of fetal neural stem cells into the glial cell effect of EPO.

**Conclusion:**

EPO treatment immediately after OGD/R could not facilitate fetal NSCs/NPs neurogenesis but promoted the formation of the EPOR/βCR heterodimer on fetal NSCs/NPs, which mediates its function in glial differentiation.

## Graphical abstract

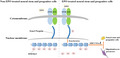

## 1 INTRODUCTION

Erythropoietin (EPO) is a cytokine mainly induced under hypoxic conditions, which primarily acts on erythroid progenitor cells in the bone marrow. Compelling evidence has revealed EPO's secondary functions, with a crucial focus on the central nervous system (CNS). High‐dose EPO administration prompts newly differentiating neurons in the adult mouse brain.^1^ However, the regulatory effects of EPO on neuronal differentiation at different stages of brain development remain unclear.

EPO may interact with up to four distinct isoforms of its receptor (homodimers EPOR/EPOR or hetero‐oligomers EPOR/βCR), activating different signaling cascades with roles in neuroprotection and neurogenesis. High‐dose EPO administration amplifies autocrine/paracrine EPO/EPOR signaling, prompts the emergence of new CA1 neurons, and enhances dendritic spine densities.^1^ Although the presence of these different isoforms paves the way for new interesting mechanisms through which EPO could exert its function in the brain, many questions remain unanswered. Certainly, it is necessary to investigate how EPO's binding to each isoform activates a specific intracellular pathway, modulating the expression of target genes. Once these mechanisms have been elucidated, it will be possible to develop new isoform‐selective drugs, resulting in a more specific therapy. However, the direct interaction between EPOR and βCR to form an innate repair receptor remains controversial,^2^ and whether EPOR/βCR heterodimer is present on NSCs/NPs and whether it mediates the neurogenesis effect of EPO remain unknown.

This study used primary cortical NSCs/NPs from fetal mice to explore the effect of EPO intervention on neurogenesis in vitro at different time points after reoxygenation and the possible mechanisms to provide a novel scientific basis for future experimental studies and clinical trials of cerebral ischemic diseases.

## 2 MATERIALS AND METHODS

### 2.1 Culture of primary neural stem cells (NSCs) and neural progenitors (NPs)

Female C57BL/6J mice (25–30 g, 2‐month‐old) were purchased from Vital River Laboratory Animal Technology and approved by the Institutional Animal Care and Use Committee of Capital Medical University. Cortices from embryonic day 14 mice were isolated and minced in a 0.25% trypsin solution for 5 min at 37°C. The tissue was washed in media containing 10% fetal bovine serum and then passed through a 70‐μm cell strainer. The extracted cells were resuspended in complete medium containing Dulbecco's Modified Eagle's medium (DMEM)/F12, neurobasal, 2% B27, 10 ng/ml epidermal growth factor (EGF), 10 ng/ml basic fibroblast growth factor (bFGF), 1% GlutaMAX, and 1% penicillin/streptomycin, plated in ultra‐low attachment six well‐plates, and maintained at 37°C in a humidified atmosphere containing 5% CO_2_. NSCs and NPs were subcultured by Accutase every 2–3 days. The third passage of cells was plated onto PDL‐coated cell culture plates with complete medium.

### 2.2 Oxygen and glucose deprivation (OGD) operation

The cell medium was replaced with glucose‐free DMEM (Gibco; Thermo Fisher Scientific, Inc.) in a three‐gas incubator containing 95% nitrogen and 5% CO_2_ at 37°C for 1, 2, 4, 6, and 8 h. The cells were then switched to glucose‐containing medium and maintained at 37°C in a 5% CO2 incubator for 1, 2, 4, and 7 days.

### 2.3 Cell viability assay

Cell viability was assessed using the CellTiter‐Glo® Luminescent Cell Viability Assay. Briefly, the plate and its contents were equilibrated at room temperature for approximately 30 min, followed by the addition of a volume of CellTiter‐Glo®Reagent equal to the volume of cell culture medium present in each well and mixed for 2 min on an orbital shaker to induce cell lysis. The plate was incubated at room temperature for 10 min to stabilize the luminescent signal, and luminescence was recorded using a luminometer.

### 2.4 EdU incorporation assay

Proliferation of NSCs and NPs was assessed using Click‐iT® EdU Imaging Kits according to the manufacturer's instructions. Isolated NSCs and NPs were seeded in 48‐well‐plates (4 × 10^5^ cells/well) and cultured in medium without EGF and bFGF. After OGD operation, EdU was added to the medium, and the cells were cultured for another 4 days. Cultured cells were fixed with 4% paraformaldehyde (PFA) for 15 min, followed by permeabilization using 0.5% Triton X‐100. After permeabilization, the cells were incubated with Click‐iT® reaction cocktail for 30 min in a dark room. The total number of cells and Edu‐positive cells were counted using high‐content analysis.

### 2.5 Flow cytometry for epidermal growth factor receptor and doublecortin assessment

Neurospheres were dissociated using Accutase (Sigma‐Aldrich). A fraction of cells from each tube was selected to prepare a negative control tube (unmarked cells). Cells were fixed with 4% PFA for 30 min, washed with 1 ml phosphate‐buffered saline (PBS) 0.15% bovine serum albumin (BSA), and incubated with 0.5% Triton X‐100 for 30 min. Cells were washed once with 1 ml PBS 0.15% BSA and incubated with EGF receptor (EGFR) antibody (Alexa Fluor® 750, Novus) or doublecortin (DCX) antibody (PE, Biorbyt) for 30 min in the dark. The cells were washed once and resuspended in PBS. Cells were then analyzed on a BD FACSCalibur flow cytometer.

### 2.6 Quantitative real‐time reverse transcription PCR

Purified RNA from NSCs and NPs was used as a template to synthesize cDNA using oligo‐d (T) primers and SuperScript III /RNaseOUT Enzyme Mix (Invitrogen). Relative gene expression was calculated using the 2^−ΔΔ*C*T^ method, normalized, and expressed as fold change relative to U6 or β‐actin. Real‐time polymerase chain reaction was performed in triplicate: primers for myelin basic protein (*Mbp*): F: 5’‐TCCGACGAGCTTCAGACCA‐3′ and R: 5’‐ACCCCTGTCACCGCTAAAGA‐3′, primers for 2′,3′‐cyclic nucleotide 3′‐phosphodiesterase (*CNPase*): F:5’‐GCCTTCAAGAAAGAGCTTCG‐3′ and R: 5’‐CAGATCACTGGGCCACAACT‐3′, primers for microtubule‐associated protein‐2 (*Map‐2*): F: 5’‐TCTCTAAAGAACATCCGTCAC‐3′ and R: 5’‐ATCTTCACATTACCACC TCC‐3′, primers for β‐tubulin‐III: F:5’ GCGCCTTTGGACACCTATT‐3′ and R:5’‐CCAGCACCACTCTGACCAA‐3′, primers for glial fibrillary acidic protein (*GFAP*): F: 5’‐AACAACCTGGCTGCGTAT‐3′ and R: 5’‐CTGCCTCGTATTGAGTGC‐3′, primers for S100 calcium‐binding protein B: F:5’‐CCCTCATTGATGTCTTCCACC3’ and R:5’‐TTCCTGCTCCTTGATTTCCTC‐3′, primers for *Epor*: F:5’‐TCCTGGAGCACCTAT GACC‐3′ and R:5’‐CGAGATGAGGACCAGAATGA‐3′, and primers for βcr (*Csf2rb*): F:5’‐TGGAGCAAGTGGAGCGAA‐3′ and R:5’‐CACAGCCAAAGCGAAGGAT‐3′.

### 2.7 Co‐immunoprecipitation

Co‐immunoprecipitation (Co‐IP) assays were performed to identify the proteins. Briefly, 1 × 10^6^ cells were collected and lysed in 300 μl buffer containing non‐denaturing lysis buffer, protease inhibitor, and phosphatase inhibitors. For Co‐IP using antibodies, before being added to the cell lysates, the antibodies were incubated with Protein A/G Magnetic Beads (Sigma‐Aldrich) and IgG for 3 h at 4°C to eliminate nonspecific binding. Subsequently, the cross‐linked Protein A/G Magnetic Beads were added directly to the cell lysates and incubated overnight at 4°C. The magnetic beads were washed with IP wash buffer and collected. The protein complexes were eluted from the beads using 50 mM glycine (pH 2.8) and analyzed by western blotting.

### 2.8 Liquid chromatography tandem mass spectrometry

Protein bands were excised from the sodium dodecyl sulfate–polyacrylamide gel electrophoresis gels and were dissolved with 50 μl mobile phase A (H_2_O, 0.1% formic acid) and loaded onto an Acclaim PepMap C18‐reversed‐phase column (75 μm × 2 cm, 3 μm, 100 Ǻthermo Scientific) and separated with reversed‐phase C18 column (75 μm × 10 cm, 5 μm, 300 Ǻ Agela Technologies) mounted on a Dionex ultimate 3000 nano LC system. Peptides were eluted using a gradient: starting from 5% Buffer B; 0–6 min 5%–8% Buffer B; 6–40 min 8%–30% Buffer B; 40–45 min 30%–60% Buffer B, 45–48 min 60%–80% Buffer B; 48–56 min 80% Buffer B; 56–58 min increasing to 5% Buffer B; 58–65 min 5% Buffer B at a flow rate of 400 nL/min combined with a Q–Exactive mass spectrometer (Thermo Fisher Scientific). The eluates were directly entered Q–Exactive MS (Thermo Fisher Scientific), setting in positive ion mode and data‐dependent manner with full MS scan from 350 to 2000 m/z, full scan resolution at 70,000, and MS/MS scan resolution at 17,500. The MS/MS scan had a minimum signal threshold 1E+5 and isolation width of 2 Da. To evaluate the performance of this mass spectrometry on the samples, two MS/MS acquisition modes and higher collision energy dissociation (HCD) were employed. To optimize the MS/MS acquisition efficiency of HCD, the normalized collision energy was systemically examined as 28%.

### 2.9 Western blotting

Cell samples were processed for western blotting analysis, as previously described. The poly(vinylidene fluoride) membranes (Millipore Corporation) were incubated with the following primary antibodies at 4°C overnight: anti‐EPOR (rabbit, orb164257, Biorbyt, 1:1000), anti‐βCR (mouse, 393,281, Santa, 1:1000), and anti‐H3K9me3 (mouse, 5327, CST, 1:100). Antigen–antibody complexes were observed by enhanced chemiluminescence using an Immobilon Western blotting kit. The intensity of the bands was detected using a FluorChem®HD2 Gel Imaging System (Protein Simple). The gray values of the bands were analyzed using AlphaEase FC software (Alpha Innotech).

### 2.10 β common receptor siRNA transfection

NSCs and NPs were transfected with βCR siRNA using Lipofectamine Stem Transfection Reagent (Invitrogen) for 48 h according to the manufacturer's instructions, and the transfection efficiency was validated by western blotting.

### 2.11 Immunofluorescence analysis

The cells were fixed in 4% PFA for 15 min. After washing three times in PBS, the cells were incubated with 3% BSA for 1 h. Cells were immunostained with primary antibodies against EPOR (rabbit, orb164257; Biorbyt, 1:100), anti‐βCR (mouse, 393,281; Santa, 1:100), anti‐H3K9 (mouse, 5327; CST, 1: 100), and anti‐Syne‐1 (rabbit, ab192234; Abcam, 1:100). After incubation with secondary antibodies conjugated with DyLight 488 or Cy3 (Jackson ImmunoResearch), the cells were counterstained with 4′,6‐diamidino‐2‐phenylindole. The images were digitized using an Olympus Fluoview FV1000 microscope (Olympus).

### 2.12 Statistical analysis

Statistical analysis was performed using SPSS version 23.0. Data were tested for normality using the Shapiro–Wilk test (*p* < 0.05). Numerical data are presented as the mean standard deviation. Student's *t*‐test was used for two‐group comparisons. One‐way analysis of variance (ANOVA) with the Tukey–Kramer post hoc test was used for comparisons among several quantitative variables. The correlation between the two variables was assessed using the Pearson's correlation test. Data for neurobehavioral tests were analyzed using two‐way repeated measures ANOVA followed by the Bonferroni post hoc correction. The correlation between two variables was determined using Pearson's correlation test. *p* < 0.05 was considered statistically significant.

## 3 RESULTS

### 3.1 OGD time‐dependently depressed neurogenesis of NSCs/NPs by decreasing cell proliferation and viability

**FIGURE 1:**
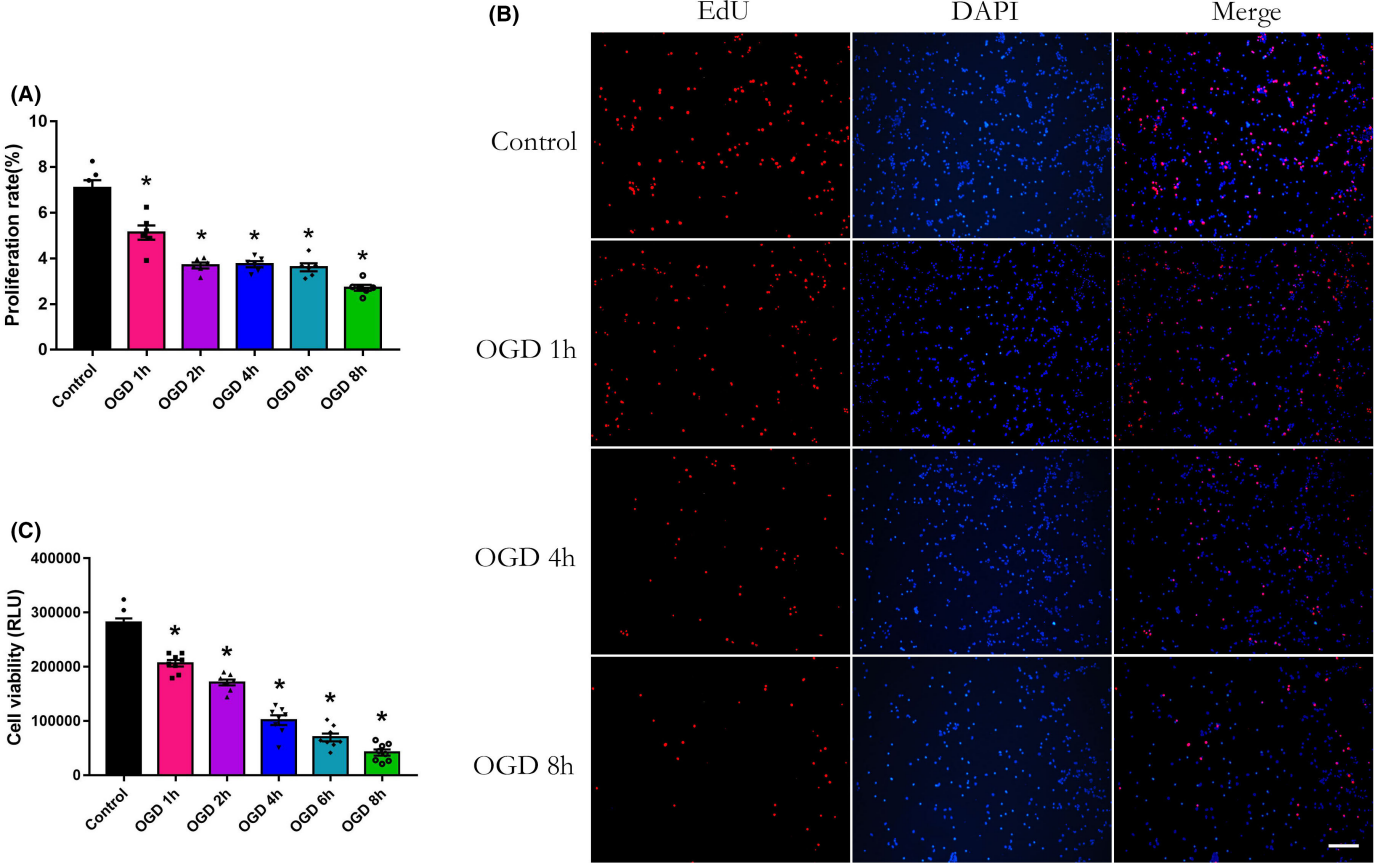
Cell proliferation and viability of neural stem and progenitor cells were decreased after oxygen–glucose deprivation/reoxygenation (OGD/R) treatment. (A, B) Proliferation rate was detected at OGD/R4d by Edu analysis. The positive (red) cells indicated activated proliferating cells. Scale bar: 30 μm. *n* = 6/group. (C) Cell viability was measured at OGD/R2d by CellTiter‐Glo®Luminescent assay. Control, OGD1h, 2 h, 4 h, 6 h, and 8 h, respectively, denote cells without and with 1, 2, 4, 6, and 8 h OGD treatment. *n* = 8/group. *p < 0.05 vs. the control group

To mimic the pathological changes in hypoxic–ischemic injury, we isolated cerebral cortical NSCs/NPs from fetal mice and established the OGD model and investigated the direct effect of hypoxic injury on the proliferation rate and cell viability of NSCs/NPs by Edu incorporation and CellTiter‐Glo cell viability assay. The results indicated that OGD significantly reduced both the proliferation (Figure 1A, B, *p* < 0.05) and cell viability (Figure 1C, *p* < 0.05) of NSCs/NPs. Furthermore, the cell viability decreased continuously with the extension of hypoxic time (Figure 1B, *p* < 0.05), whereas the decrease in cell proliferation plateaued from 2 h to 6 h (Figure 1A, *p* < 0.05), suggesting that the 2 h to 6 h might be a mild duration time of the OGD model on the NSCs/NPs. In summary, we showed that hypoxia damaged neurogenesis, partly by decreasing cell viability and proliferation.

### 3.2 EPO treatment at different time point after OGD/R rescued cell viability rather than proliferation of NSCs/NPs

**FIGURE 2:**
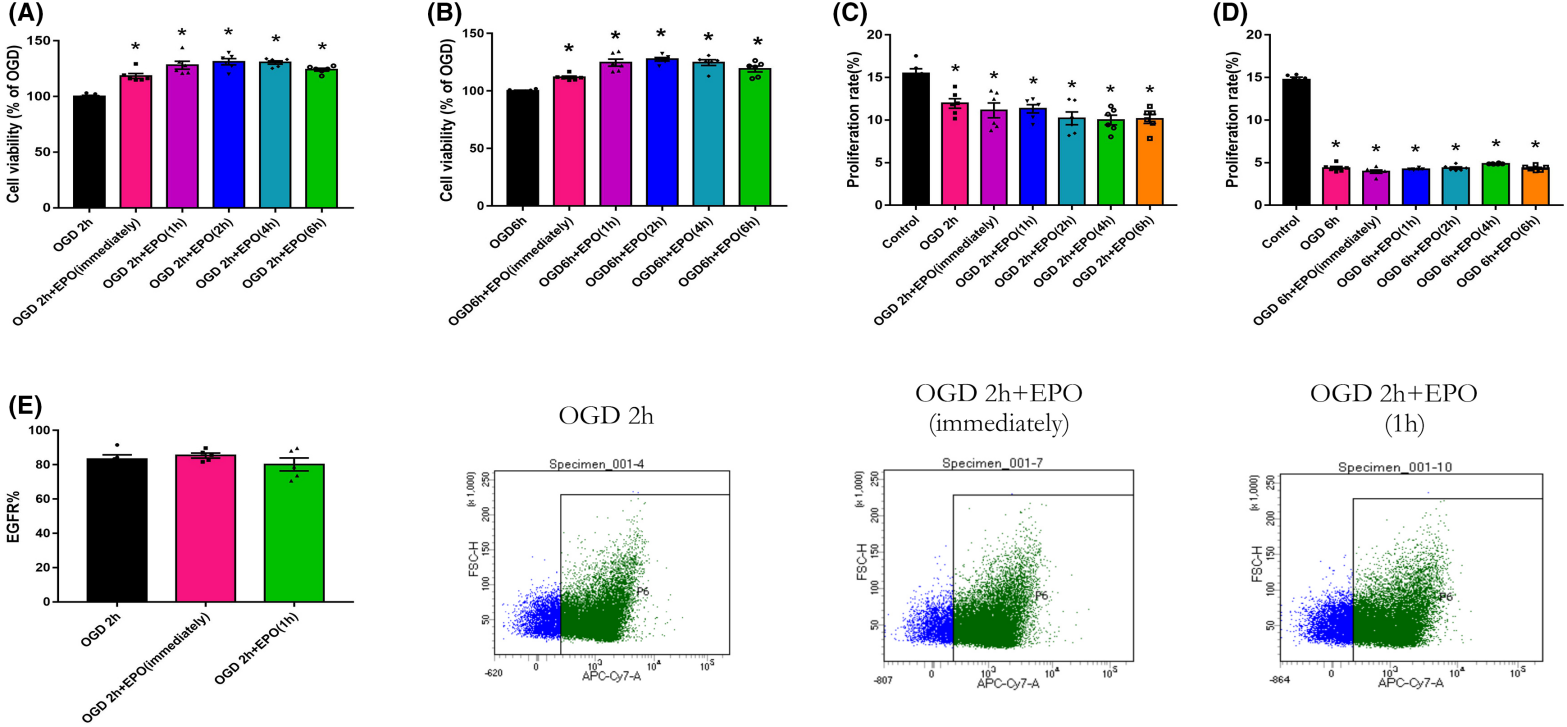
EPO increases the cell viability while exerting no effect on proliferation of neural stem and progenitor cells. (A, B) Cell viability after 2 or 6 h of OGD plus EPO treatment was determined by CellTiter‐Glo®Luminescent assay at 2 days after OGD. *n* = 6/group. *p < 0.05 vs. the OGD group. (C, D) Cell proliferation rate after 2 and 6 h of OGD plus EPO treatment was determined by Edu assay at 4 days after OGD. EPO (immediately) denotes cells were given EPO (50 U/ml) immediately after OGD treatment. EPO (1, 2, 4, 6 h) denotes cells were given EPO (50 U/ml) 1, 2, 4, or 6 h after OGD treatment. *n* = 6/group. *p < 0.05 vs. the control group. (E) EGFR% was detected by flow cytometry 1 day after 2 h of OGD treatment. OGD denotes cells were not given EPO treatment after OGD. EPO (immediately) denotes cells were given EPO (50 U/ml) immediately after OGD treatment. EPO (1 h) denotes cells were given EPO (50 U/ml) 1 h after OGD treatment. *n* = 6/group

Next, we explored the effect of EPO on the proliferation rate and cell viability of cortical NSCs/NPs after OGD 2 and 6 h, representing mild and severe hypoxic injury. We showed that EPO administration at 1 to 6 h after OGD for 2 or 6 h could increase the survival of NSCs/NPs (Figure 2A, B, *p* < 0.05), with no effect on the proliferation rate (Figure 2C, D). Moreover, we detected EGFR% by flow cytometry to compare the ratio of actively proliferating cells among the 2 h OGD, immediate EPO treatment after OGD (EPO immediately), and delayed EPO treatment (EPO 1 h) groups. The results indicated that there was almost no difference in the ratio of EGFR‐positive cells among the three groups 24 h after OGD (Figure 2E). Therefore, immediate or delayed delivery of EPO could promote neural regeneration after hypoxic injury by increasing cell viability without influencing cell proliferation.

### 3.3 EPO treatment immediately after OGD/R boosted the differentiation of NSCs/NPs toward oligodendrocytes and astrocytes

**FIGURE 3:**
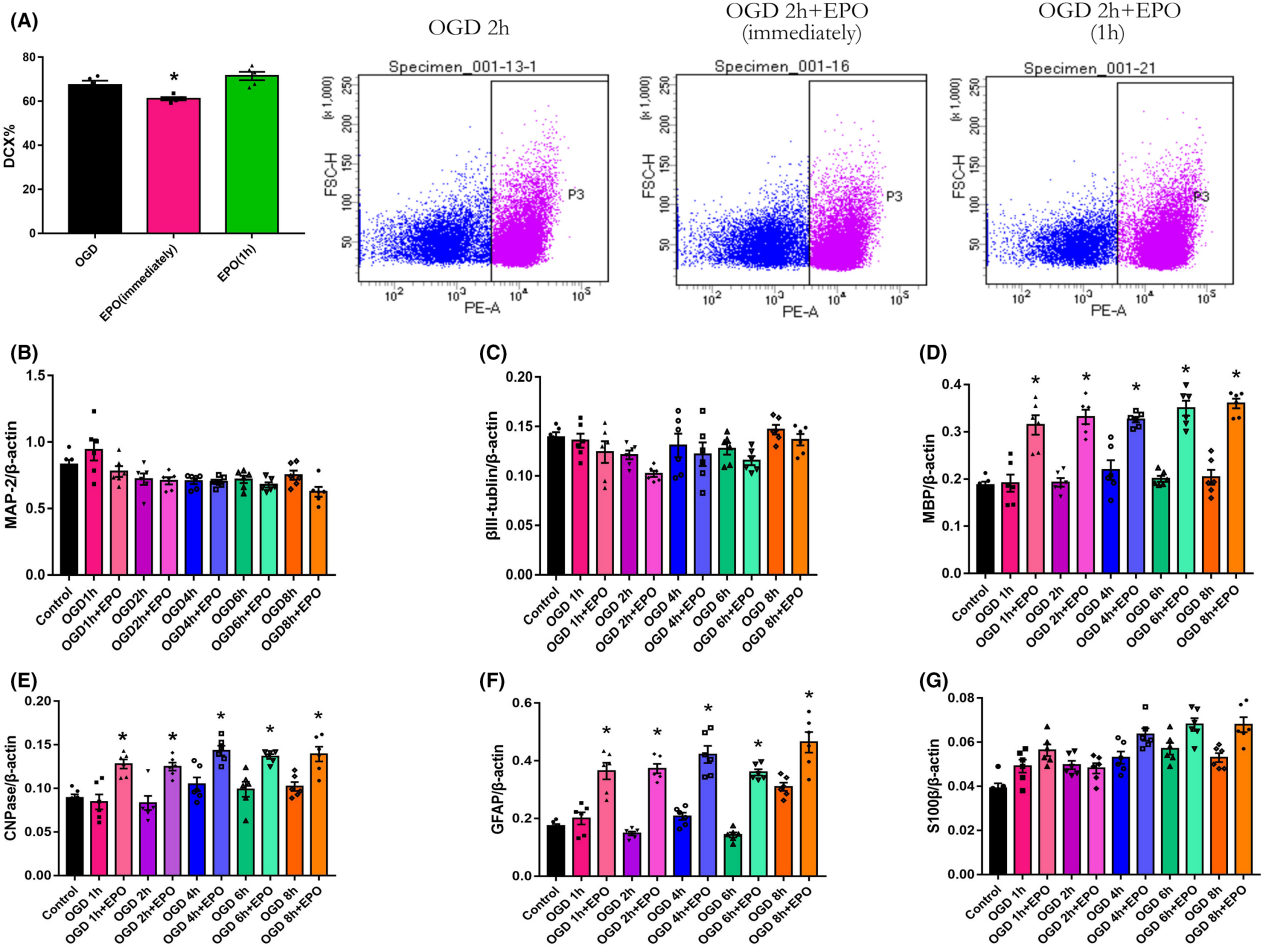
EPO treatment increases the differentiation of neural stem and progenitor cells toward oligodendrocytes and astrocytes at 7 day after OGD/R. (A) DCX% was detected by flow cytometry 1 day after 2 h of OGD treatment. OGD denotes cells were not given EPO treatment after OGD. EPO (immediately) denotes cells were given EPO (50 U/ml) immediately after OGD treatment. EPO (1 h) denotes cells were given EPO (50 U/ml) 1 h after OGD treatment. *n* = 6/group. *p < 0.05 vs. the OGD group. Gene expression of neuronal markers MAP‐2 (B) and βIII‐tubulin (C), the oligodendrocyte markers MBP (D) and CNPase (E), astrocyte markers GFAP (F) and S100β (G) in different groups were determined by RT‐PCR. Control denotes cells without OGD treatment. OGD 1, 2, 4, 6 and 8 h denote cells were treated with 1, 2, 4, 6 and 8 h OGD. OGD 1, 2, 4, 6 and 8 h + EPO denote cells were given 50 U/ml EPO after OGD operation for 1, 2, 4, 6 and 8 h. *n* = 6/group. *p < 0.05 vs. the control group

Differentiation of NSCs/NPs into neurons, oligodendrocytes, and astrocytes is another key process during neurogenesis. First, we explored differentiation toward neurons. We detected DCX% by flow cytometry to compare the differentiation to neurons in the 2 h OGD, immediate EPO treatment after OGD (EPO immediately), and delayed EPO treatment (EPO 1 h) groups 1 day after OGD 1, 2, 4, 6, and 8 h (Figure 3A). Unexpectedly, when treated immediately, EPO decreased the DCX%, indicating the inhibition of EPO on differentiation toward neurons (Figure 3A, *p* < 0.05), whereas EPO treatment 1 h after OGD did not alter the ratio of DCX‐positive cells, suggesting that delayed delivery of EPO exerts no effect on the differentiation of NSCs/NPs toward neurons. Furthermore, by detecting the relative mRNA levels of MAP‐2 and β‐tubulin, two markers for mature neurons, at 7 days after OGD, we found that OGD and immediate EPO treatment did not significantly alter the potential of NSCs/NPs to differentiate into neurons (Figure 3B, C).

We further investigated the effect of immediate EPO treatment on the glial differentiation of NSCs/NPs. We demonstrated that OGD from 1 to 8 h did not significantly change the relative mRNA levels of MBP and CNPase, two markers of mature oligodendrocytes, 7 days after OGD, suggesting that the differentiation toward oligodendrocytes may not be directly affected by hypoxia. However, the relative mRNA levels of both MBP and CNPase were significantly elevated after EPO administration, suggesting that EPO could promote oligodendrocyte regeneration (Figure 3D, E, *p* < 0.05). The mRNA level of GFAP, a marker for mature astrocytes, was significantly increased by EPO treatment. Another marker for astrocytes, S100β, was also slightly increased by EPO treatment in the OGD 1, 4, 6, and 8 h groups, indicating that EPO could also promote the differentiation toward astrocytes (Figure 3F, G, *p* < 0.05). In summary, EPO treatment immediately after reoxygenation promoted the differentiation of NSCs/NPs toward oligodendrocytes and astrocytes, but not neurons.

### 3.4 EPOR/βCR heterodimer exists on NSCs/NPs

**FIGURE 4:**
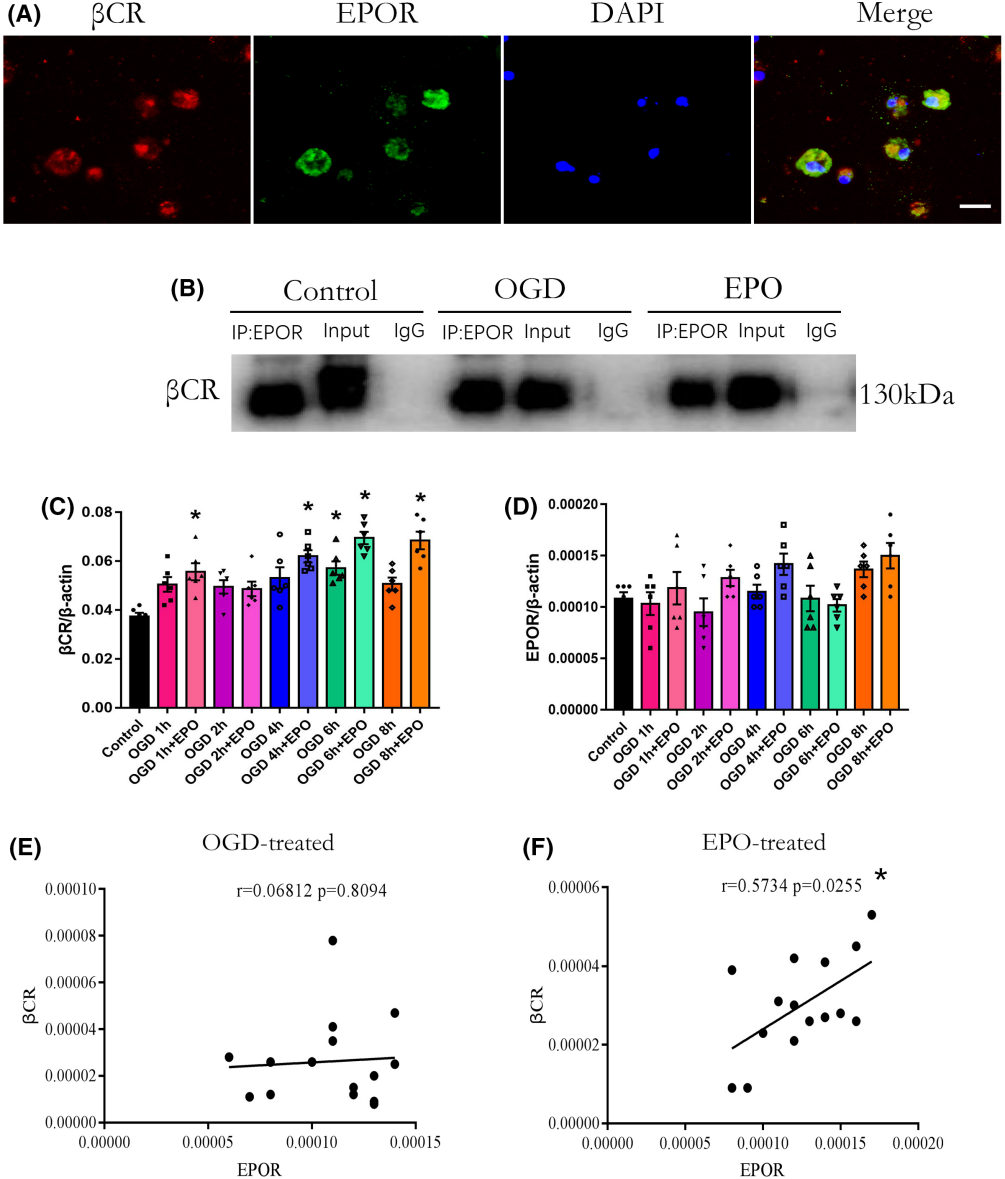
EPOR/βCR heterodimer exists on primary neural stem and progenitor cells. (A) Confocal microscopy was used to determine the colocalization of EPOR (green) and βCR (red) on neural stem and progenitor cells. Nuclei were stained with DAPI (blue). Scale bar: 5 μm. (B) Cellular lysates immunoprecipitated (IP) with anti‐EPOR antibody (Co‐IP) or mouse lgGb2 (lgG) and cell lysates without IP treatment (input) were immunoprobed with anti‐βCR antibody. (C, D) Gene expression of the EPOR and βCR in different groups of cells was measured by RT‐PCR. Control denotes cells without OGD treatment. OGD 1, 2, 4, 6 and 8 h denote cells were treated with 1, 2, 4, 6 and 8 h OGD. OGD 1, 2, 4, 6 and 8 h + EPO denote cells were given 50 U/ml EPO after OGD operation for 1, 2, 4, 6 and 8 h. *n* = 6/group. *p < 0.05 vs. the control group. (E, F) Correlation between EPOR and βCR after OGD or plus EPO treatment

To determine the existence of EPOR homodimers or hetero‐oligomers on the surface of NSCs/NPs, immunofluorescence, confocal microscopy, and Co‐IP were used. We first identified that both EPOR/EPOR homodimers and EPOR/βCR hetero‐oligomers were present on the NSCs/NPs (Figure 4A). Furthermore, Co‐IP indicated that EPOR and βCR were bound together in NSCs/NPs in the control, OGD, and EPO treatment groups (Figure 4B). Moreover, EPO treatment did not influence the mRNA levels of EPOR (Figure 4D), but increased the expression of βCR in NSCs/NPs compared with the control group (Figure 4C, *p* < 0.05). Correlation analysis revealed that the mRNA levels of βCR were positively correlated with the mRNA levels of EPOR when cells were treated with EPO after OGD (Figure 4F, *p* < 0.05), whereas cells without EPO treatment after OGD showed no significant correlation between EPOR and βCR (Figure 4E). In summary, we preliminarily showed the existence of an EPOR/βCR heterodimer on the surface of NSCs/NPs, and EPO treatment significantly increased the mRNA expression of βCR and elevated the correlation between EPOR and βCR levels.

### 3.5 Correlation between EPOR/βCR and neural markers in NSCs/NPs cells

**FIGURE 5:**
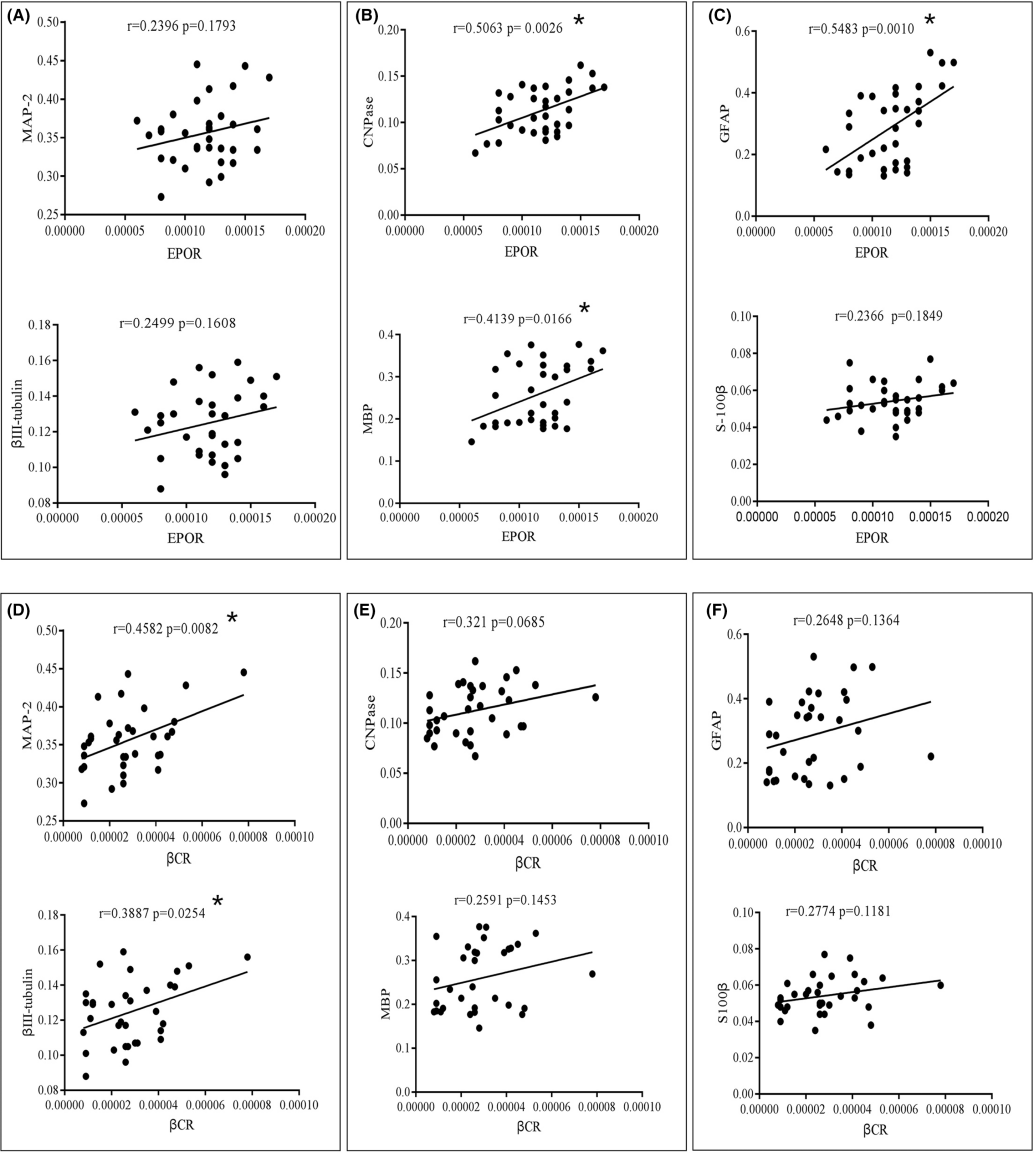
Correlation between EPOR/βCR and neural markers in primary neural stem and progenitor cells. (A) Correlation between EPOR and neuronal markers MAP‐2 and βIII‐tubulin. (B) Correlation between EPOR and oligodendrocyte markers MBP and CNPase. (C) Correlation between EPOR and astrocyte markers GFAP and S100β. (D) Correlation between βCR and neuronal markers MAP‐2 and βIII‐tubulin. (E) Correlation between βCR and oligodendrocyte markers MBP and CNPase. (F) Correlation between βCR and astrocyte markers GFAP and S100β

To establish a preliminary association between EPOR/βCR hetero‐oligomers and neural cell markers, we performed a correlation analysis between the mRNA levels of cell markers and the two receptors of EPO. The results indicated that EPOR was positively correlated with the expression of CNPase, MBP, and GFAP (Figure 5B, C, *p* < 0.05), whereas no significant trends were observed between EPOR and MAP‐2, βIII‐tubulin, and S100β (Figure 5A, C). βCR was positively correlated with the expression of MAP‐2 and βIII‐tubulin (Figure 5D, *p* < 0.05), and no significant correlation was found between βCR and CNPase, MBP, GFAP, and S100β (Figure 5E, F). Our results suggest that EPOR might be involved in the differentiation of NSCs/NPs into mature oligodendrocytes and astrocytes, whereas βCR is possibly related to the differentiation of NSCs/NPs into mature neurons. Therefore, the expression of EPOR/βCR heterodimer was correlated with the expression of oligodendrocyte markers.

### 3.6 Syne‐1 (Nesprin) binds to EPOR/βCR heterodimer and βCR mediated Syne‐1/H3K9 signaling downstream of EPO

**FIGURE 6:**
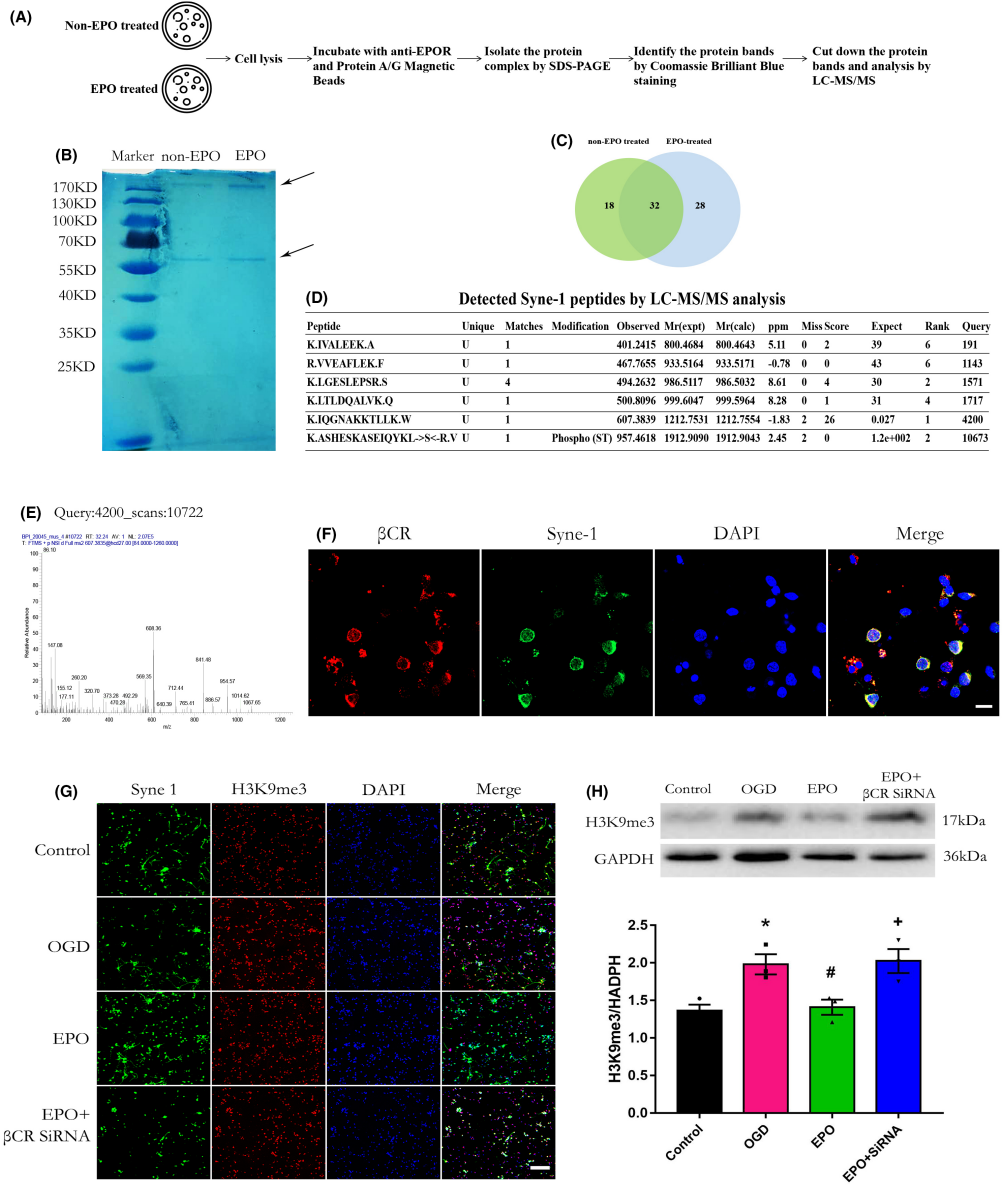
Syne‐1/H3K9me3 signaling pathway mediated the effect of EPO through EPOR/βCR heterodimer in primary neural stem and progenitor cells. (A) The flowcharts of LC–MS/MS analysis. (B) Coomassie brilliant blue staining of the SDS‐PAGE gel. The arrowhead indicates the protein bands. (C) Venn analysis of protein bands specifically pulled down by EPOR/βCR heterodimer from non‐EPO‐treated and EPO‐treated (EPO) neural stem and progenitor cell lysates. (D) The table of Syne‐1 peptides identified by LC–MS/MS. (E) The ion mass spectra of one matched peptide of Syne‐1. (F) Primary neural stem and progenitor cells were immunostained with βCR (red) and Syne‐1 (green) for confocal microscopy examination. Nuclei were stained with DAPI (blue). Scale bar: 5 μm. (G) Confocal microscopy was used to determine the colocalization of Syne‐1 (green) and H3K9me3 (red). Nuclei were stained with DAPI (blue). Scale bar: 50 μm. Cells were treated with βCR siRNA for 48 h and were then treated with EPO (50 U/ml) immediately after OGD. (H) Protein expression level of H3K9me3 was assessed by western blotting. n = 3/group. *p < 0.05 vs. the control group, ^#^ *p* < 0.05 vs. the OGD group, ^+^ *p* < 0.05 vs. the EPO group

To explore the possible downstream signaling pathways of EPOR/βCR hetero‐oligomers, LC–MS/MS was used to analyze the EPOR‐interacting proteins (Figure 6A). Two protein bands were pulled down by the EPOR antibody after nonspecific binding proteins were removed by IgG and Protein A/G magnetic beads (Figure 6B), and LC–MS/MS analysis was performed. We identified 50 and 60 potential EPOR‐binding proteins from OGD‐ and EPO‐treated cells, respectively. Venn analysis showed that 32 of these proteins were commonly found in two groups of cells (Figure 6C), and 18 and 28 proteins (including four predicted and characterized proteins) were found only in OGD‐ and EPO‐treated cells, respectively (Table 1). In addition, there were five potential proteins that were oxidized (Table 2) and another five potential proteins that were phosphorylated (Table 3) in the EPO‐treated cells. In sum, we identified 37 types of potential proteins that were expressed or chemically modified by EPO treatment, with one potential protein, Syne‐1, which was among the 28 proteins found only in EPO‐treated cells and phosphorylated by EPO treatment (Figure 6D, E). Among them, we hypothesized that Syne‐1 is a key molecule for the effect of EPO on the oligodendrocyte differentiation of NSCs/NPs based on a previous study.^3^ Since Syne‐1 was found to bind to EPOR, confocal immunofluorescence showed that Syne‐1 was colocalized with βCR on the NSCs/NPs (Figure 6F), suggesting that Syne‐1 is a potential downstream signaling molecule of the EPOR/βCR heterodimer.

**TABLE 1:** Proteins which were found only in EPO‐treated cells

Gene name	Description	Mass	Score	Matches	Sequence	emPAI	Coverage
Actg2	Actin, gamma‐enteric smooth muscle (Fragment) OS = Mus musculus OX = 10,090 GN = Actg2 PE = 3 SV = 1	22,158	60	3 (2)	1 (1)	0.15	8%
Tuba1a	Tubulin alpha‐1A chain OS = Mus musculus OX = 10,090 GN = Tuba1a PE = 1 SV = 1	50,788	49	6 (3)	4 (2)	0.13	12%
Ralgapb	Ral GTPase‐activating protein subunit beta OS = Mus musculus OX = 10,090 GN = Ralgapb PE = 1 SV = 1	168,180	22	14 (0)	3 (0)	0.02	0%
Eif4g1	Eif4g1 protein (Fragment) OS = Mus musculus OX = 10,090 GN = Eif4g1 PE = 2 SV = 1	93,878	15	2 (0)	2 (0)	0.03	2%
Syne1	Nesprin‐1 OS = Mus musculus OX = 10,090 GN=Syne1 PE = 1 SV = 1	1,016,647	26	9 (1)	6 (1)	0	0%
Fgf10	Fibroblast growth factor 10 OS = Mus musculus OX = 10,090 GN=Fgf10 PE = 2 SV = 1	24,095	23	2 (0)	1 (0)	0.14	3%
Pde11a	Dual 3′,5′‐cyclic‐AMP and ‐GMP phosphodiesterase 11A OS = Mus musculus OX = 10,090 GN=Pde11a PE = 1 SV = 1	105,523	21	1 (0)	1 (0)	0.03	1%
Igkv4‐92	Immunoglobulin kappa variable 4–92 (Fragment) OS = Mus musculus OX = 10,090 GN=Igkv4‐92 PE = 4 SV = 1	12,882	17	1 (0)	1 (0)	0.27	13%
Cltc	Clathrin heavy chain OS = Mus musculus OX = 10,090 GN=Cltc PE = 1 SV = 1	193,631	81	4 (2)	4 (2)	0.03	3%
Atp1a2	Sodium/potassium‐transporting ATPase subunit alpha OS = Mus musculus OX = 10,090 GN = Atp1a2 PE = 1 SV = 1	104,717	62	2 (1)	2 (1)	0.03	2%
Vdac3	Voltage‐dependent anion‐selective channel protein 3 OS = Mus musculus OX = 10,090 GN=Vdac3 PE = 1 SV = 1	31,175	47	1 (1)	1 (1)	0.11	4%
Plxna3	Plexin A3 OS = Mus musculus OX = 10,090 GN=Plxna3 PE = 4 SV = 1	211,361	41	5 (1)	2 (1)	0.02	0%
Pkm	Pyruvate kinase PKM (Fragment) OS = Mus musculus OX = 10,090 GN=Pkm PE = 1 SV = 1	20,809	39	1 (1)	1 (1)	0.16	5%
Top2b	DNA topoisomerase 2‐beta OS = Mus musculus OX = 10,090 GN = Top2b PE = 1 SV = 2	182,707	34	5 (1)	4 (1)	0.02	2%
Dnajc8	DnaJ homolog subfamily C member 8 OS = Mus musculus OX = 10,090 GN=Dnajc8 PE = 1 SV = 1	25,819	26	2 (0)	1 (0)	0.13	5%
Nop56	Nucleolar protein 56 (Fragment) OS = Mus musculus OX = 10,090 GN=Nop56 PE = 1 SV = 1	14,164	24	6 (0)	2 (0)	0.24	11%
H2bc14	Histone H2B OS = Mus musculus OX = 10,090 GN=H2bc14 PE = 1 SV = 1	13,928	24	5 (1)	2 (1)	0.25	19%
Ap5z1	AP‐5 complex subunit zeta‐1 OS = Mus musculus OX = 10,090 GN = Ap5z1 PE = 1 SV = 1	88,721	22	3 (0)	1 (0)	0.08	1%
Try5	Peptidase S1 domain‐containing protein OS = Mus musculus OX = 10,090 GN = Try5 PE = 2 SV = 1	28,002	18	2 (0)	1 (0)	0.12	7%
Cyp4a30b	Cytochrome P450, family 4, subfamily a, polypeptide 30b OS = Mus musculus OX = 10,090 GN=Cyp4a30b PE = 3 SV = 1	58,952	16	1 (0)	1 (0)	0.06	1%
Tspyl2	Testis‐specific Y‐encoded‐like protein 2 OS = Mus musculus OX = 10,090 GN = Tspyl2 PE = 1 SV = 1	77,737	16	1 (0)	1 (0)	0.04	1%
Cfap57	Cilia‐ and flagella‐associated protein 57 OS = Mus musculus OX = 10,090 GN=Cfap57 PE = 1 SV = 3	145,756	24	2 (1)	2 (1)	0.02	1%
Rp1l1	MCG56960 OS = Mus musculus OX = 10,090 GN = Rp1l1 PE = 2 SV = 1	202,299	23	2 (0)	1 (0)	0.02	0%
Ppp1r9a	Protein phosphatase 1, regulatory subunit 9A OS = Mus musculus OX = 10,090 GN=Ppp1r9a PE = 1 SV = 1	109,834	14	1 (0)	1 (0)	0.03	0%

*Note*: Mass: protein molecular weight; Score: protein score; Matches: the total number of matched peptides; Sequence: the total number of sequences matched; emPAI: 10^(N observed/N observable)−1^; Coverage: Protein identification coverage.

**TABLE 2:** Proteins which were oxidized only in EPO‐treated cells

Gene name	Description	Mass	Score	Matches	Sequence	emPAI	Coverage
Hba‐x	Hemoglobin X, alpha‐like embryonic chain in Hba complex (Fragment) OS = Mus musculus OX = 10,090 GN=Hba‐x PE = 1 SV = 1	18,092	34	2 (1)	2 (1)	0.19	13%
Vim	Vimentin OS = Mus musculus OX = 10,090 GN=Vim PE = 1 SV = 1	49,220	60	4 (2)	2 (1)	0.07	5%
H2bc14	Histone H2B OS = Mus musculus OX = 10,090 GN=H2bc14 PE = 1 SV = 1	13,928	24	5 (1)	2 (1)	0.25	19%
Cyp4a30b	Cytochrome P450, family 4, subfamily a, polypeptide 30b OS = Mus musculus OX = 10,090 GN=Cyp4a30b PE = 3 SV = 1	58,952	16	1 (0)	1 (0)	0.06	1%
Atp5f1b	ATP synthase subunit beta, mitochondrial OS = Mus musculus OX = 10,090 GN = Atp5f1b PE = 1 SV = 2	56,265	82	2 (2)	2 (2)	0.12	4%

*Note*: Mass: protein molecular weight; Score: protein score; Matches: the total number of matched peptides; Sequence: the total number of sequences matched; emPAI: 10^(N observed/N observable)−1^; Coverage: Protein identification coverage.

**TABLE 3:** Proteins which were phosphorylated only in EPO‐treated cells

Gene name	Description	Mass	Score	Matches	Sequence	emPAI	Coverage
Ralgapb	Ral GTPase‐activating protein subunit beta OS = Mus musculus OX = 10,090 GN = Ralgapb PE = 1 SV = 1	168,180	22	14 (0)	3 (0)	0.02	0%
Cyp4a30b	Cytochrome P450, family 4, subfamily a, polypeptide 30b OS = Mus musculus OX = 10,090 GN=Cyp4a30b PE = 3 SV = 1	58,952	16	1 (0)	1 (0)	0.06	1%
Syne1	Nesprin‐1 OS = Mus musculus OX = 10,090 GN=Syne1 PE = 1 SV = 1	1,016,647	26	9 (1)	6 (1)	0.00	0%
Hba‐x	Hemoglobin X, alpha‐like embryonic chain in Hba complex (Fragment) OS = Mus musculus OX = 10,090 GN=Hba‐x PE = 1 SV = 1	18,092	34	2 (1)	2 (1)	0.19	13%
Fgf10	Fibroblast growth factor 10 OS = Mus musculus OX = 10,090 GN=Fgf10 PE = 2 SV = 1	24,095	23	2 (0)	1 (0)	0.14	3%

*Note*: Mass: protein molecular weight; Score: protein score; Matches: the total number of matched peptides; Sequence: the total number of sequences matched; emPAI: 10^(N observed/N observable)−1^; Coverage: Protein identification coverage.

According to previous studies, H3K9me3 is one of the downstream molecules of Syne‐1 in oligodendrocyte progenitors, and the methylation of H3K9 exerts important effects on myelination and cell differentiation.^3^, ^4^, ^5^ Thus, we investigated whether Syne‐1/H3K9me3 is downstream of the EPOR/βCR heterodimer. Immunofluorescence staining showed that Syne‐1 expression was decreased after OGD, whereas EPO treatment increased Syne‐1 expression, which was reversed by βCR siRNA (Figure 6G). Furthermore, western blotting demonstrated that H3K9me3 was upregulated after OGD and was reduced by EPO treatment. Similarly, βCR siRNA reversed the effect of EPO on H3K9me3 (Figure 6H, *p* < 0.05). Thus, EPO activates the EPOR‐βCR/Syne‐1/H3K9me3 signaling pathway and controls the cell fate switch of NSCs/NPs.

## 4 DISCUSSION

The present study investigated the effect of EPO on post‐hypoxic neurogenesis using primary cortical NSCs/NPs in fetal mice. We demonstrated that EPO treatment at different time points after OGD/R could promote cell viability while exerting no significant effect on cell proliferation. EPO treatment immediately after OGD/R boosted the differentiation of NSCs/NPs toward oligodendrocytes and astrocytes. Moreover, EPOR/βCR heterodimer existed on the cell surface of the fetal cortical NSCs/NPs, and the Syne‐1/H3K9me3 signaling pathway probably mediated the effect of EPOR/βCR heterodimer on oligodendrocytes. Collectively, our main contribution revealed that EPO treatment immediately after OGD/R could not promote neurogenesis, and the existence of EPOR/βCR heterodimer on fetal NSCs/NPs mediated its function in glial differentiation.

Enhancement of neurogenesis is generally an effective therapeutic strategy for brain injury, especially hypoxia/ischemia‐induced brain damage. As a prominent neuroprotective agent, many preclinical experiments have shown that EPO can promote neurogenesis and oligodendrogenesis following hypoxic–ischemic cerebral insults. However, the present study showed that EPO treatment increased the viability of fetal cortical NSCs/NPs and promoted their proliferation and differentiation toward oligodendrocytes and astrocytes rather than neurons. After cerebral stroke, endogenous neurogenesis was insufficient to restore the damaged neurological function, which was partly due to the high apoptosis rate of NSCs/NPs.^6^ One mechanism of EPO on the generation of mature erythrocytes is to increase cell viability by activating the PI3K signaling pathway or the Bcl‐2 family.^7^, ^8^ Previous studies have suggested that EPO administration could increase the proliferation and neuronal differentiation of normal NSCs/NPs.^9^ Conversely, our results indicated that instead of cell proliferation and neuronal differentiation, EPO could promote cell viability and differentiation toward oligodendrocytes and astrocytes of NSCs/NPs following OGD/R, which is partly consistent with previous results of Hassouna that EPO may be able to increase neurogenesis without entering the cell cycle.^10^ The white matter of the CNS comprises myelin and is derived from oligodendrocytes.^11^ Hypoxia produces a rapid and significant loss of axons in both the acute and subacute periods.^12^ Spontaneous axonal regeneration is fundamental but inadequate for restoring function. Therefore, our results show that EPO treatment immediately after OGD/R cannot directly promote the differentiation of NSCs/NPs into neurons but can reduce the axonal damage of neurons by promoting the differentiation of oligodendrocytes and astrocytes. Our data showed that EPO treatment immediately after OGD/R was not a good time point for neurogenesis, further demonstrating that the effects of EPO are dose‐ and time‐dependent in the hypoxic‐injured brain.

EPOR/βCR exists in primary human renal epithelial cells, endothelial progenitor cells, and macrophages.^13^, ^14^, ^15^ At least three versions of the EPO receptor are now known to exist within the brain, including the βCR.^16^ Although the heteromeric EPO receptor involving the βCR was initially hypothesized to confer neuroprotection, evidence now supports a role for this heteromeric EPO receptor and the homodimeric EPO receptor in the protection of neurons and glia.^16^ The nonpeptidyl compound STS‐E412, a type of EPO that selectively binds to the EPOR/βCR heterodimer, could act as a neuroprotective agent in the CNS, suggesting the neuroprotective role of the EPOR/βCR heterodimer.^13^ Our results demonstrated for the first time that EPOR/βCR heterodimers also exist on primary NSCs/NPs. Moreover, EPO treatment increased the mRNA level of βCR and the correlation between EPOR and βCR. In contrast to our current findings, it was previously shown that EPOR expression is induced by hypoxia and EPO, and its distribution corresponds to that of EPO, suggesting that brain EPO works in a paracrine/autocrine manner in response to hypoxia. Our data suggest that EPO could promote the formation of the EPOR/βCR heterodimer, and the effect of EPO on NSCs/NPs was mediated by the EPOR/βCR heterodimer. Additionally, βCR was elevated by EPO treatment, whereas the differentiation toward neurons was not altered by EPO, demonstrating that there may be ligands other than EPO that mediate the effect of βCR on neural differentiation, which needs further studies.

We also performed a correlation analysis to further identify the possible association between EPOR/βCR and neural cell differentiation. EPOR was demonstrated to be associated with the oligodendrocyte markers MBP and CNPase and the astrocyte marker GFAP, suggesting that it might be involved in oligodendrocyte and astrocyte differentiation. βCR was correlated with the neuronal markers β‐tubulin and MAP‐2, suggesting that βCR is possibly involved in neuronal differentiation. EPO treatment immediately after OGD/R boosted the differentiation of NSCs/NPs toward oligodendrocytes and astrocytes. Therefore, EPOR/βCR heterodimer expressions were correlated with oligodendrocyte marker expression.

A better understanding of the progression of NSCs/NPs in the developing cerebral cortex is important for modeling neurogenesis. To further identify the downstream signaling of the EPOR/βCR heterodimer, we analyzed the protein bands isolated by Co‐IP with EPOR antibody and found several types of protein fragments that were likely to be involved in the effect of EPO on NSCs/NPs, among which Syne‐1 (nesprins) might be related to oligodendrocyte differentiation.^3^ Syne‐1 is a family of multi‐isomeric scaffolding proteins that form the linker of nucleoskeleton‐and‐cytoskeleton with SUN (Sad1p/UNC84) proteins at the nuclear envelope. Syne‐1 is extensively expressed in the adult murine CNS and regulates axon termination and synapse formation during neurodevelopment in the nervous system.^17^, ^18^ The N‐terminal cytosolic domain of Syne could act as a connection with the C‐terminal domain of cytosolic actin to interact with the nuclear periphery and with nucleosomal histones carrying repressive marks, indicating its important role in transducing signals into the nucleus.^19^ Histone‐3 lysine 9 (H3K9) is one of the downstream repressive histones of Syne‐1 in mouse oligodendrocyte progenitor differentiation.^3^ H3K9 is associated with pericentric heterochromatin and is important for genomic stability, and trimethylation at H3K9 (H3K9me3) is enriched in an adult NSC niche.^4^ H3K9me3 is enriched in genes associated with cellular maintenance and is not expressed in axon/neuron generation,^4^ indicating that epigenetic modification (H3K9me3) is involved in cell fate transition. In addition, H3K9 demethylation is related to cell differentiation.^5^ We found that the expression of H3K9me3 in the NSCs/NPs increased after OGD treatment, whereas EPO treatment decreased the level of H3K9me3, which might be associated with the effect of EPO on the oligodendrocyte differentiation of NSCs/NPs.

The current study has some limitations. A recent study showed that cell–cell interactions may exert neuroprotective effects on ischemic stroke in in vitro experiments.^20^, ^21^, ^22^ Moreover, some studies have strongly suggested that glial cells, such as microglia, protect against neuronal cell death in cultures.^23^, ^24^ Our previous study investigated the neuroprotective effects of EPO by shifting microglial polarization and inhibiting excessive gliogenesis after cerebral ischemia in mice.^25^ Certainly, this study also missed opportunities to explore the crosstalk between microglia and NSCs/NPs because only pure NSCs/NPs were used in the current experiments. Therefore, more stereoscopic studies should be conducted in future studies.

In summary, our results demonstrated that EPO treatment immediately after OGD/R could increase cell viability and differentiation toward oligodendrocytes of primary fetal mouse cortical NSCs/NPs. The EPOR/βCR heterodimer and its downstream signaling pathway, Syne‐1/H3K9, were possibly involved in the effect of EPO on the oligodendrocyte differentiation of NSCs/NPs.

## AUTHOR CONTRIBUTIONS

Z.Y., S.Z., H.Z., F.L., and Z.T. conducted the study design, experiments, data analysis, and manuscript preparation. Y.L. and R.W. designed and managed the study.

## CONFLICT OF INTEREST

The authors declare no conflicts of interest.

## Data Availability

The data that support the findings of this study are available from the corresponding author upon reasonable request.
